# Simultaneous Fitting of Absorption Spectra and Their Second Derivatives for an Improved Analysis of Protein Infrared Spectra

**DOI:** 10.3390/molecules200712599

**Published:** 2015-07-10

**Authors:** Maurizio Baldassarre, Chenge Li, Nadejda Eremina, Erik Goormaghtigh, Andreas Barth

**Affiliations:** 1 Department of Biochemistry and Biophysics, Stockholm University, 10691 Stockholm, Sweden; E-Mails: maurizio.baldassarre@dbb.su.se (M.B.); chenge.li@dbb.su.se (C.L.); naer2045@dbb.su.se (N.E.); 2 Center for Structural Biology and Bioinformatics, Université Libre de Bruxelles, 1050 Brussels, Belgium; E-Mail: egoor@ulb.ac.be

**Keywords:** Fourier-transform infrared spectroscopy, FT-IR, curve fitting, amide I, secondary structure, second derivative

## Abstract

Infrared spectroscopy is a powerful tool in protein science due to its sensitivity to changes in secondary structure or conformation. In order to take advantage of the full power of infrared spectroscopy in structural studies of proteins, complex band contours, such as the amide I band, have to be decomposed into their main component bands, a process referred to as curve fitting. In this paper, we report on an improved curve fitting approach in which absorption spectra and second derivative spectra are fitted simultaneously. Our approach, which we name co-fitting, leads to a more reliable modelling of the experimental data because it uses more spectral information than the standard approach of fitting only the absorption spectrum. It also avoids that the fitting routine becomes trapped in local minima. We have tested the proposed approach using infrared absorption spectra of three mixed α/β proteins with different degrees of spectral overlap in the amide I region: ribonuclease A, pyruvate kinase, and aconitase.

## 1. Introduction

For several decades infrared spectroscopy has been an invaluable tool in the study of protein structures, conformations, and dynamics. One of its main applications is the analysis of protein secondary structure. Here, the usefulness of infrared spectroscopy originates from its ability to detect changes in relative orientation and hydrogen-bonding of the peptide C=O groups, which are the main structure-related oscillators. C=O oscillators on different peptide groups couple, which leads to delocalized normal modes. The absorption band collectively arising from these groups, known as the amide I band (approx. 1700–1620 cm^−1^), is the sum of the absorption of all normal modes. Depending on the type of secondary structure in which the C=O groups are involved, the normal modes tend to cluster within specific spectral regions, giving rise to component bands, each with its own spectral parameters (position, bandwidth and extinction coefficient). The component bands make it possible to perform qualitative and quantitative studies on the secondary structure of a protein [1,2,3,4,5]. One of the most significant, practical advantages for a detailed structural analysis is that the amide I band is sensitive to more than the mere secondary structure. Other structural parameters can alter the amide I spectrum, such as solvent exposure [6,7], local deformations [2,8], size [2], and structural organization [9,10,11,12,13] of the individual secondary structure elements. These phenomena increase the inherent information content, but complicate spectral interpretation. They can be exploited to monitor biological processes, even those that do not lead to substantial net changes in the secondary structure content, including local conformational changes [14,15], association/dissociation [16,17] and binding of substrates or ligands [15,18,19]*.*

The amide I band often shows a complex, unresolved line shape where the maximum is accompanied by a variable number of shoulders. A pivotal aspect in the study of proteins by means of infrared spectroscopy becomes, then, to identify and characterize the main component bands underneath the amide I profile in terms of position, width, and intensity or area. This is necessary to estimate the secondary structure content of a protein. Curve fitting represents the most common approach to obtain such information [4,20]. The basic principle of curve fitting is to reconstruct the original spectrum, or part of it, using a defined set of bands for which all or some of the parameters are allowed to change [21]. Some initial parameters, such as the number and positions of the component bands, must be assessed beforehand from resolution enhanced spectra. Resolution enhancement (also referred to as band narrowing) is a mathematical analysis of the absorption spectrum which enhances narrow features in the spectrum. This leads to a seeming improvement of the spectral resolution. Approaches for resolution enhancement are for example: calculation of the second or fourth derivative [22,23] of an absorption spectrum, Fourier self-deconvolution [24], and fine-structure enhancement [2].

The rationale of any curve fitting procedure is to minimize the differences between the experimental absorption spectrum and the reconstructed (or fitted) spectrum. The current approach is to take into account only the absorption spectrum. However, even a good match between the fitted and the experimental spectra is no guarantee for good agreement between their resolution enhanced spectra, as pointed out previously [3]. An alternative to fitting the absorption spectrum is to fit resolution enhanced spectra. Here it was pointed out previously that Fourier deconvoluted spectra or Fourier derivatives should be fitted with Fourier manipulated bands [25]. A corresponding approach is to fit derivatives of component bands to the experimental derivative spectrum [26,27]. This should not be confused with an evaluation of band areas of second derivative spectra by integration [28] or fitting of what seem to be Gaussian lines [29].

Fitting of resolution-enhanced spectra has been questioned for a number of reasons [30]. Firstly, resolution-enhanced spectra are more affected by instrumental noise and water vapor contamination than absorption spectra. Because water vapor bands do not have a constant intensity in the amide I range, but rather a bell-shaped distribution centered at ~1650 cm^−1^, this could create a hidden, intrinsic bias towards bands in that region. Secondly, derivation over-represents sharper bands over broader ones, making some structural features, e.g., random coils, virtually invisible. The first criticism is no longer valid, since it is possible nowadays to record high quality resolution-enhanced spectra if sufficient care is taken. The obvious solution to the second problem is to fit absorption and derivative spectra simultaneously. This was suggested previously and applied to spectra in the visible spectral range [31]. The approach has also been used to analyze the amide I band of the protein Ca^2+^-ATPase but the procedure was manual and time-consuming [32].

In this work, we describe the use of a combined fitting approach whereby the differences between the experimental and fitted absorption spectra as well as between the corresponding second derivatives are minimized. We name our approach co-fitting and note that it is different from the previous evaluation or fitting of only the second derivative spectra or Fourier-deconvoluted spectra. The results are compared to those obtained upon fitting the absorption spectrum only according to common practice, which is termed standard fitting further on. The effects of improving the agreement between the second derivatives is assessed.

Apart from synthetic curves, we have used three proteins in this study: ribonuclease A, pyruvate kinase, and aconitase. Ribonuclease A (bovine pancreatic ribonuclease A) is a relatively small (124 residues, ~13.7 kDa), but highly stable enzyme that cleaves single-stranded RNA. The three-dimensional structure of ribonuclease A (Figure 2) shows three short *N*-terminal α-helices surrounded by two β-sheets, and an overall secondary structure composition of 20% α-helices and 33% β-sheets [33]. Rabbit muscle pyruvate kinase is a large tetrameric mitochondrial protein (530 residues and ~58.0 kDa/monomer) containing four domains: a short, mostly α-helical *N*-terminal domain, a mixed α/β barrel domain (a parallel β-barrel including eight strands surrounded by eight α-helices), an antiparallel β-barrel domain, and a C-terminal, mixed α/β domain [34]. The overall secondary structure composition is 38% α-helices and 20% β-sheets. Aconitase is a large (754 residue, ~85.7 kDa) mitochondrial enzyme that catalyzes the stereospecific dehydration/rehydration of citrate to isocitrate in the Krebs cycle. The crystal structure (Figure 2) shows high α-helical content (34.6%) and a mixed population of β-sheets (19.5%) arranged in two slightly twisted, parallel β-sheets and a mixed parallel/anti-parallel β-barrel [35].

## 2. Results and Discussion

### 2.1. Method Benchmarking and Quality Assessment

**Figure 1:**
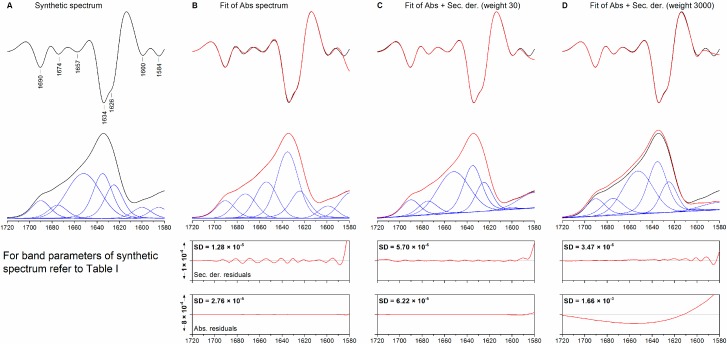
(**A**) Synthetic spectrum obtained using the spectral parameters defined in Table 1. (**B**) Fitting of the absorption spectrum only; (**C**–**D**) simultaneous fitting of the absorption and second derivative spectra using the same set of initial spectral parameters and weight factors of 30 and 3000. From top to bottom: second derivative, absorption spectra, residuals for the second derivative spectrum, and residuals for the absorption spectrum. Original spectra are shown black, fitted spectra in red. The individual bands are shown in blue.

The simultaneous fitting of an absorption spectrum and its second derivative (co-fitting) was tested for its ability to fit a synthetic spectrum of a hypothetical protein composed of overlapping bands with mixed Gaussian-Lorentzian line shapes. Although all fitting procedures described in this paper were performed between 1720 and 1580 cm^−1^, additional bands were added to the synthetic spectra below 1580 cm^−1^, including a broad band that represents the amide II absorption. To mimic a real-case scenario, the following actions were undertaken: (1) statistical noise was added to the synthetic spectra to yield a noise level similar to that observed in our experimental spectra; (2) the initial positions of the bands used for fitting were determined from the minima in the second derivative spectra; (3) a dynamic baseline was allowed during fitting although the synthetic spectra did not contain any baseline. Figure 1 shows the synthetic spectrum (panel A), as well as the results of standard fitting and co-fitting using different weights (panels B–D). Because second derivative spectra are significantly less intense than their original absorption spectra, they need to be multiplied by an appropriate scaling factor, or weight, in order to influence the outcome of co-fitting. A low weight means that the agreement between absorption spectra is favored, while a high weight favors agreement between second derivative spectra. A weight of 3000, as used in panel D, approaches the case where only the second derivative spectrum is fitted. Spectral parameters are reported in Table 1.

Fitting the absorption spectrum only (standard fitting), a high agreement between the fitted absorption spectrum and the real spectrum (SD_Abs_ = 2.76 × 10^−5^), as well as between the corresponding second derivatives (SD_Der_ = 1.28 × 10^−5^), can be observed. In this fit, the baseline is equal to 0 throughout the fitting range. Comparison of the individual fitted bands (panel B) with the original bands (panel A) reveals, however, significant discrepancies. For instance, the low-frequency β-sheet band has a higher contribution in the fitted spectrum than in the real spectrum (area +9%), while the broad (width 35 cm^−1^) band arising from irregular and α-helical structures, originally present in the synthetic spectrum at 1652 cm^−1^ is lost in favor of a weaker (area −21%) and sharper (width 23 cm^−1^) band at approx. the same position. This is compensated by an overestimation (+4%) of the band arising from hypothetical turn-like structures at 1675 cm^−1^.

**Table 1:** Band parameters of a synthetic spectrum and of fitted spectra obtained upon standard fitting and co-fitting. Values in italics correspond to bands representing amide II and tyrosine vibrations which are outside the fitting range. The fg values denote the fractional contribution of the Gaussian lineshape to the overall lineshape. The initial value was 0.50 for all bands.

Band	Initial Position/cm^−1^	Final Position/cm^−1^	Width (fg)	Intensity	Area/%
***Parameters for Synthetic Spectrum***					
1	1690		20 (0.7)	0.008	9.3
2	1675		20 (0.7)	0.006	7.1
3	1652		35 (1.0)	0.020	37.5
4	1635		20 (0.7)	0.020	23.6
5	1625		19 (0.7)	0.015	16.8
6	1600		20 (0.7)	0.005	5.7
7	1585		20 (0.7)	0.005	4.3
*8*	*1550*		*45 (1.0)*	*0.025*	
*9*	*1515*		*15 (0.7)*	*0.002*	
***Fitting to Abs spectrum only***					
1	See above	1690.5	19.9 (0.63)	0.008	8.4
2		1672.7	23.2 (1.00)	0.011	11.5
3		1653.9	22.9 (1.00)	0.016	16.9
4		1635.1	22.6 (0.83)	0.030	32.7
5		1624.3	20.0 (0.55)	0.012	13.4
6		1599.1	19.6 (1.00)	0.006	4.9
7		1579.0	23.7 (1.00)	0.011	12.3
***Fitting to Abs + Der, 30***					
1	See above	1690.1	18.9 (0.81)	0.007	8.1
2		1675.0	19.9 (0.75)	0.006	7.4
3		1652.0	33.5 (1.00)	0.019	33.5
4		1635.0	19.5 (0.74)	0.021	24.4
5		1625.0	17.0 (0.85)	0.013	12.7
6		1600.5	11.3 (0.61)	0.001	0.6
7		1579.0	35.1 (1.00)	0.007	13.3
***Fitting to Abs + Der, 3000***					
1	See above	1690.1	20.1 (0.68)	0.008	9.44
2		1674.8	22.0 (0.63)	0.008	10.17
3		1652.6	31.6 (1.00)	0.019	31.51
4		1635.2	20.3 (0.68)	0.023	27.67
5		1625.1	17.3 (0.88)	0.014	12.93
6		1600.5	12.9 (0.13)	0.001	0.93
7		1579.0	41.6 (1.00)	0.003	7.34

Using co-fitting and a weight of 30, the agreement between the fitted and real absorption spectra is decreased (SD_Abs_ = 6.22 × 10^−5^) in favor of a better agreement between the corresponding second derivative spectra (SD_Der_ = 5.70 × 10^−6^). Although a baseline is introduced during fitting, the shape of the individual fitted bands and their relative intensities are strikingly similar to the original values. The areas of the bands at 1675 and 1635 cm^−1^ are predicted with an error below 1%. In addition to this, their line shape, described by the contribution of the Gaussian component to the overall band (0.75 and 0.74), is similar to the original value of 0.7. The random coil band is similar (−4%), in terms band area, to the original band, and so are its width (33.5 cm^−1^, original value 35 cm^−1^) and line shape (calculated and original values are both 1). Larger divergences are observed for the weaker bands outside the amide I range. Using a much higher weight for co-fitting (3000) implies practically that only the second derivative spectrum is fitted. This fit yields results similar to the previous case, although the agreement between the fitted bands and the real bands in the amide I region is generally lower than with a weight of 30.

These results show that co-fitting can provide significant advantages over standard fitting and can yield more accurate results when fitting spectra with overlapping bands. This improvement is brought about by only a slight improvement of the fit to the second derivative spectrum in a case where standard fitting already gave a very good agreement between the second derivatives of original spectrum and fit. The results show also that small differences in the agreement between the second derivatives can lead to significant changes in the parameters of the fitted bands.

In order to challenge the co-fitting approach, we generated similar spectra in which a small, narrow (15 cm^−1^) and a strong, broad (35 cm^−1^) band overlapped near 1650 cm^−1^. They mimicked contributions from α-helices and irregular structures respectively. In these cases, only one band could be identified in the second derivative spectrum, which was largely dominated by the narrow band. Consequently, the fit model contained one band less than the original model. We expected that co-fitting should detect that a band is missing in the fit model, but found that it produced a very good fit to both the second derivative and the original spectrum in spite of the missing band. To further test this, the fitting was repeated with an additional band allowed to lie anywhere between 1700 and 1600 cm^−1^. Even in this favorable case, however, the fitting algorithm was unable to identify the co-existence of the broad band at 1652 cm^−1^ and the sharp band at 1655 cm^−1^. For this reason, fitting of the experimental spectra described throughout this work was performed with the same number of bands that could be identified in second derivative spectra (or fourth derivative spectra, in case of ambiguities). Therefore, special attention should be paid when fitting spectra of proteins containing significant contributions of both α-helix and irregular structures. This setback is likely to be avoided in cases were total hydrogen-deuterium exchange is possible. This leads to a relatively larger downshift of the signal from irregular conformations. However, when hydrogen-deuterium exchange is only partial, the band pattern is likely more complex and an interpretation may be more difficult. In summary, co-fitting seems to improve the fit when the component bands can be resolved, but it does not necessarily “rescue” situations where the fit model cannot be determined from the experimental data.

### 2.2. Overview of the Protein Infrared Spectra

**Figure 2:**
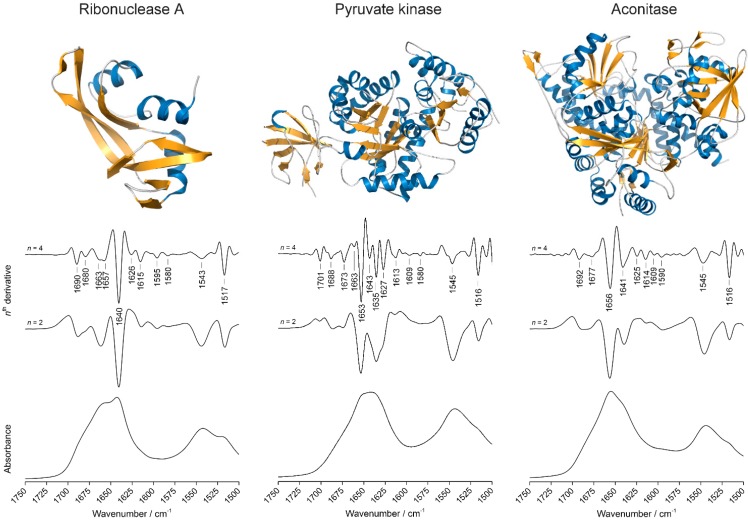
Three-dimensional structures, infrared absorption and derivative spectra (second and fourth) of ribonuclease A, pyruvate kinase, and aconitase. α-Helices and β-sheets are colored blue and orange, respectively. Irregular structure and turns are colored grey. Spectra from bottom to top: absorption, second derivative, and fourth derivative. Second derivative spectra are scaled arbitrarily. Fourth derivative spectra are scaled and multiplied by –1. The peak absorbance values in the absorption spectra of the three proteins are 0.40, 0.29 and 0.28 for ribonuclease A, pyruvate kinase, and aconitase respectively. The largest signals in the respective second derivative spectra are 2.4 × 10^−3^, 1.0 × 10^−3^, and 1.1 × 10^−3^ cm^2^. The PDB files used to generate the three-dimensional structures are, in the order, 5RSA, 2G50, and 1B0J.

Figure 2 shows the three-dimensional structures, the absorption spectra and the second and fourth derivative spectra for the following proteins: ribonuclease A (left), pyruvate kinase (center), and aconitase (right). In the spectral window displayed (1750–1500 cm^−1^), the absorption spectrum of each protein is dominated by two broad bands originating from molecular vibrations involving the peptide bond. The amide I band (approx. 1700–1620 cm^−1^) originates mainly from the stretching vibration of the C=O group, with a lesser contribution from the out-of-phase C–N stretching and the C_α_–C–N deformation vibrations. The amide II band (approx. 1550 cm^−1^) is a less “pure” one, and its intensity and frequency are due to a combination of several types of vibrations within the peptide group. Its main contribution is the in-plane N–H bending vibration [36]. The three proteins were selected because of their mixed α/β structures, which complicates fitting compared to proteins containing only one type of secondary structure, as well as for their size diversity. Accordingly, the fourth derivative spectra (multiplied by −1) show bands that reflect these dominating secondary structures: bands in the 1641 to 1620 cm^−1^ range for β-sheets and a sharp band between 1657 and 1653 cm^−1^ for α-helices. In the spectrum of ribonuclease A, a β-rich protein, this α-helical signal is smaller because of the lower α-helix content of this protein.

Water vapor produces a number of narrow bands between 2000 and 1300 cm^−1^. Because of their small band width, they are enhanced relative to the broader protein features in second and forth derivative spectra. Our second and fourth derivative spectra do not show distinctive features between 1750 and 1720 cm^−1^ (see Figure 2), as well as above 1750 cm^−1^ (not shown), demonstrating that the spectra are free from water vapor. Therefore the signals below 1700 cm^−1^ correspond to real secondary structure bands.

### 2.3. Introduction to Co-Fitting of Protein Spectra at the Example of Ribonuclease A

Figure 2 shows the absorption, second and fourth derivative spectra of a hydrated ribonuclease A film in MOPS buffer, pH 7. The absorption spectrum shows a finely structured amide I band, with a sharp maximum at 1642 cm^−1^ and a broad shoulder at approx. 1660 cm^−1^. Apart from the main band at 1640 cm^−1^, the second derivative spectrum reveals the presence of several shoulders underneath the amide I band, such as a broad band at 1660 cm^−1^ and a doublet at 1690 and 1680 cm^−1^. The signals at 1640 and 1626 cm^−1^, together with the weaker one at 1690 cm^−1^, can be assigned to antiparallel β-sheets, while the band at 1680 cm^−1^, together with the band at 1663 cm^−1^ detected in the fourth derivative spectrum, can be assigned to turns, in agreement with previous observations available in the literature [37]. The band at 1657 cm^−1^ can be assigned to α-helices, together with a small contribution from random coils and unstructured conformations, while the weaker signal at 1615 cm^−1^ may arise from amino acid side chains, such as those of Tyr or Asn [38]. Other bands outside though in proximity of the amide I region, such as those at 1595 and 1580 cm^−1^, can arise from the side chain absorptions of Tyr, Glu, and Asp residues. Bands outside the fitting range (1720–1580 cm^−1^) were not considered in this study.

**Figure 3:**
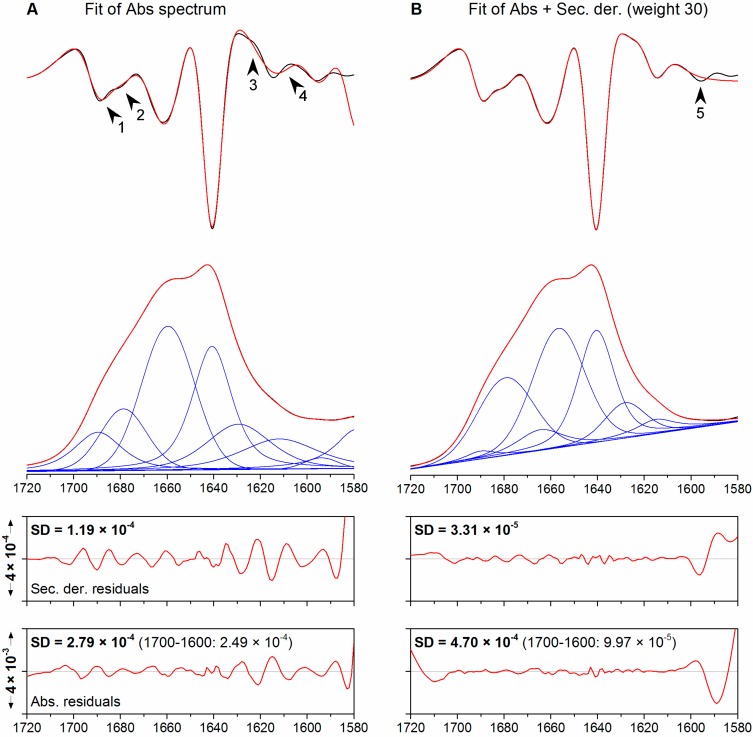
(**A**) Fitting of the absorption spectrum of ribonuclease A; (**B**) simultaneous fitting of the absorption and second derivative spectra (**B**) using the same set of initial spectral parameters and a weight factor of 30. From top to bottom: second derivative, absorption spectra and plots of the residuals. Original spectra are shown black, fitted spectra in red. The individual bands are shown in blue. Arrow heads denote noteworthy discrepancies between the experimental and fitted spectra.

Curve fitting of only the absorption spectrum, hereby referred to as *standard fitting*, of ribonuclease A was performed using the nine bands described above. Their intensities, widths and line shapes were allowed to change freely, but their positions were restricted to lie in a 10 cm^−1^ interval centered at the positions observed in the fourth derivative spectra. Fitting results are shown in Figure 3A and listed in Table 2. The final position of all bands diverged from the initial values by less than 4 cm^−1^. The fitted absorption spectrum (red trace) is entirely superimposable on the experimental spectrum (black trace), which might suggest a high accuracy for the fit. Also in the second derivative spectra, the band at 1640 cm^−1^ and the band pair at 1663/1657 cm^−1^ are superimposable. However, discrepancies can be observed for the two pairs of overlapping bands at higher and lower wavenumbers (arrowheads 1–4 in Figure 3A): in the second derivative spectrum of the fit (upper panel, red trace), only one broad band can be observed at 1615 cm^−1^ and the distinct shoulder at 1680 cm^−1^ is hardly noticeable. This suggests that these regions were not modelled correctly by the curve fitting procedure although the overall standard deviations (SD) between the fitted spectra and the experimental spectra are low (SD_Abs_ 2.79 × 10^−4^, SD_Der_ 1.19 × 10^−4^). A plot of the residuals reveals, indeed, that the best agreement between the fit and the experimental data is found near the two main bands, approx. between 1670 and 1630 cm^−1^. For both the absorption spectrum and its second derivative, the similarity of fit and experimental spectrum decreases near the edges of the fitting range, where the amplitude is relatively small.

The concomitant fitting of the absorption spectrum and the second derivative spectrum, using a weighting factor of 30 for the latter, and the same initial band parameters used previously, yields the results shown in Figure 3B and listed in Table 2. As in the previous case, the fitted absorption spectrum is, upon visual inspection, entirely superimposable on the experimental spectrum. The SD_Abs_ increased to 4.7 × 10^−4^, which suggests a lower accuracy of the fit. On the other hand, the SD_Der_ decreases to 3.31 × 10^−5^, corresponding to a 15-fold improvement. This suggests that the agreement between the second derivatives is optimized at the expense of the absorption spectra. It must be noticed, however, that the plot of the residuals between the fitted and experimental absorption spectra has a flat appearance between 1700 and 1600 cm^−1^, while diverging near the edges of the fitting range. Calculation of SD_Abs_ in the 1700 to 1600 cm^−1^ range reveals a 2.5 fold improvement brought about by co-fitting (from 2.49 × 10^−4^ to 9.97 × 10^−5^). This indicates that all the bands underneath the amide I band were modelled to greater accuracy. The second derivatives of the fitted spectrum and the experimental spectrum (panel B, red trace) show stronger correlation and better superimposition than observed upon fitting the absorption spectrum alone (panel A, red trace). The only exception is represented by the weak band at 1595 cm^−1^ (arrowhead 5).

**Table 2:** Band parameters obtained upon standard fitting and co-fitting of ribonuclease A. Values in italics correspond to amino acid side chain absorptions and where excluded from calculation of band area of the amide I band. The fg values denote the fractional contribution of the Gaussian lineshape to the overall lineshape. The initial value was 0.50 for all bands.

Band	Initial Position	Final Position	Width (fg)	Intensity	Area/%
***Fitting of Abs spectrum alone***					
1	1690	1689.6	24.2 (0.37)	0.074	10.5
2	1680	1678.6	23.5 (0.90)	0.118	13.2
3	1663	1659.6	26.5 (1.00)	0.274	33.3
4	1657	1656.2	23.5 (0.59)	0.001	0.1
5	1640	1640.7	20.1 (0.41)	0.235	27.5
6	1626	1629.1	32.5 (0.57)	0.086	15.4
*7*	*1615*	*1611.9*	*39.5 (0.02)*	*0.058*	
*8*	*1595*	*1594.0*	*16.6 (0.42)*	*0.021*	
*9*	*1580*	*1579.0*	*21.8 (1.00)*	*0.074*	
***Co-fitting (weight: 30)***					
1	Same	1690.2	15.4 (0.17)	0.015	1.8
2	as	1679.3	28.3 (1.00)	0.148	23.0
3	above	1664.6	23.1 (0.43)	0.039	6.3
4		1656.7	26.3 (0.87)	0.227	34.8
5		1640.5	18.2 (0.57)	0.213	25.4
6		1628.2	20.8 (0.72)	0.067	8.7
*7*		*1615.0*	*20.7 (0.65)*	*0.027*	
*8*		*1590.3*	*37.2 (0.00)*	*0.000*	
*9*		*1580.3*	*44.3 (0.00)*	*0.000*	

The fourth derivative of the fit reproduces the main band at 1640 cm^−1^ well already in the standard fitting approach (not shown). With co-fitting using a weight of 30, even the minor bands are very well reproduced.

While a visual inspection of the fits with and without co-fitting might indicate largely equivalent results and the final band positions are similar, all intensities, widths, and line shapes change significantly when co-fitting is included. This has implications for an evaluation in terms of secondary structure. These will be discussed in the following in more general terms, since a quantitative secondary structure analysis of the particular proteins studied is not the focus of this work. In the case of co-fitting the relative area of the low-wavenumber β-sheets bands (at 1641 and ~1629 cm^−1^) is considerably lower (34%) than in case of fitting the absorption spectrum only (43%). Part of the difference is due to a larger area of the 1626 cm^−1^ band, which is much broader in the fit to only the absorption spectrum and where there is a clear deviation between the second derivatives of fit and experimental data. This discrepancy is even larger when also the high wavenumber β-sheet band near 1690 cm^−1^ is included. It has a much larger area (11% *vs.* 2%) when only the absorption spectrum is fitted because the high wavenumber band pair (bands at 1690 and 1680 cm^−1^) is modelled differently in the two fitting approaches. DSSP analysis of the crystal structure of ribonuclease A gives a β-sheet content of 33%, *i.e.* closer to the estimation from co-fitting.

Also the 1663/1657 cm^−1^ band pair is modelled differently in the two fitting approaches. Fitting of only the absorption spectrum shifts the position of the higher wavenumber component from the region of turn absorption (above 1660 cm^−1^) into the region of α-helix absorption (1659.6 cm^−1^, see Table 2) and concentrates nearly all intensity on this band. The shift into the α-helix region “rescues” the predicted α-helix and irregular structure content from this fit and makes it similar to the co-fitting case (33.4% and 34.8% without and with co-fitting, respectively). However, this must be considered a “lucky” coincidence for the standard fitting. A larger contribution of turns would have shifted the fitted band to higher wavenumber and outside the α-helix region leading to a drastic change in the estimated α-helix content.

### 2.4. Effect of Different Weights for the Second Derivative in Co-Fitting

#### 2.4.1. Pyruvate Kinase

The outcome of co-fitting is a trade-off between the agreement of the experimental and fitted absorption spectra and the agreement of the corresponding second derivatives. The weight factor describes the importance of the fit to the second derivative spectrum relative to that to the absorption spectrum. To test the effect of different weights on the fitting results, we have used the spectrum of pyruvate kinase, which shows significant band overlap in the low-frequency β-sheet region and thus allows to compare different fitting approaches for their ability to resolve crowded spectral regions.

The fourth derivative spectrum of pyruvate kinase (Figure 2) shows the presence of three component bands in the low-frequency β-sheet absorption region, at 1643, 1635, and 1627 cm^−1^, together with weaker high-frequency components at 1701 and 1688 cm^−1^ originating from the antiparallel arrangement of some of the β-strands. The intense and sharp signal at 1653 cm^−1^ can be assigned to α-helices, while the weaker signals at 1673 and 1663 cm^−1^ can be assigned to β-turns or turn-like structures. The spectrum and its spectral assignment are in agreement with data reported in the literature [39]. The signal at 1613 cm^−1^ can, as in the case of ribonuclease A, be assigned to the side chain absorption of Tyr and Asn residues.

**Figure 4:**
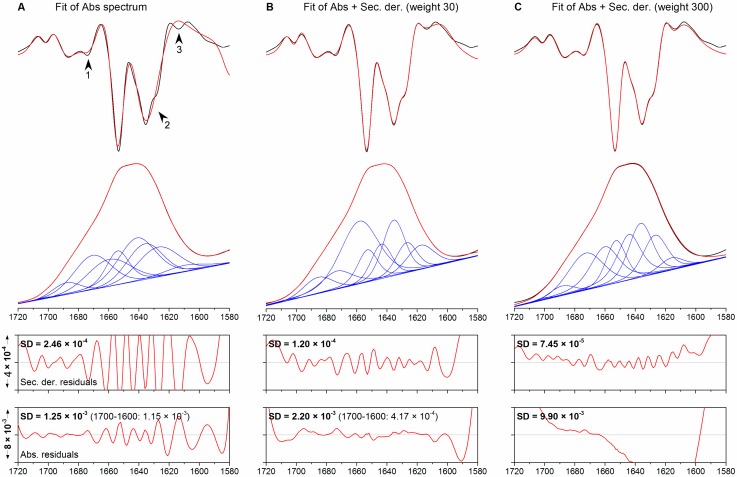
Fitting of the absorption spectrum of pyruvate kinase. (**A**) fitting of the absorption spectrum only; (**B**,**C**) simultaneous fitting of the absorption and second derivative spectra with weights of 30 and 300, respectively. From top to bottom: second derivative, absorption spectra and plots of the residuals. Original spectra are shown black, fitted spectra in red. The individual bands are shown in blue. Arrow heads denote noteworthy discrepancies between the experimental and fitted spectra.

Standard fitting of the pyruvate kinase spectrum with the ten bands detected in the fitting range produces the results shown in Figure 4A and reported in Table 3. The fitted spectrum (red trace) superimposes on the experimental spectrum (black trace) although SD_Abs_ is five times larger (1.25 × 10^−3^) than in the corresponding fit to ribonuclease A. The plot of the residuals reveals discrepancies in theα-helix/random coil and low-frequency β-sheet regions (1660–1620 cm^−1^). The second derivatives of the fit and of the experimental spectrum exhibit a markedly different line shape in the lower-frequency β-sheet region, where the three overlapping bands identified in the fourth derivative spectrum (1643, 1635 and 1627 cm^−1^) merge into a broad, single-peak band centered at 1635 cm^−1^ (arrowhead 2). This observation reveals that fitting of the absorption spectrum alone may not resolve complex patterns of overlapping bands. Other noteworthy differences between the two second derivative spectra can be observed in the signals at 1675 and 1614 cm^−1^ (arrowheads 1 and 3 in Figure 4A, respectively).

**Table 3:** Band parameters obtained upon standard fitting and co-fitting of pyruvate kinase. Values in italics correspond to amino acid side chain absorptions and where excluded from calculation of band area of the amide I band. The fg values denotes the fractional contribution of the Gaussian lineshape to the overall lineshape. The initial value was 0.50 for all bands.

Band	Initial Position	Final Position	Width (fg)	Intensity	Area/%
***Fitting of Abs spectrum alone***					
1	1701	1701.8	9.2 (0.78)	0.015	0.2
2	1688	1688.2	20.9 (1.00)	0.103	3.9
3	1673	1670.6	29.1 (0.86)	0.298	16.8
4	1663	1659.6	33.4 (1.00)	0.235	14.4
5	1653	1654.0	17.9 (0.61)	0.295	11.1
6	1643	1641.1	29.1 (0.76)	0.376	22.2
7	1635	1635.9	27.6 (0.92)	0.311	16.2
8	1627	1627.2	31.1 (0.70)	0.260	14.9
*9*	*1613*	*1613.4*	*28.6 (0.69)*	*0.062*	
*10*	*1609*	*1609.8*	*31.3 (1.00)*	*0.057*	
*11*	*1580*	*1579.0*	*27.2 (0.00)*	*0.096*	
***Co-fitting (weight: 30)***					
1	Same	1701.3	7.6 (0.94)	0.007	0.1
2	as	1685.6	22.3 (1.00)	0.141	6.3
3	above	1673.1	24.3 (0.54)	0.157	9.2
4		1658.2	29.6 (0.73)	0.545	36.2
5		1652.8	15.6 (0.63)	0.280	10.2
6		1643.7	15.7 (0.83)	0.301	10.2
7		1635.5	17.2 (0.81)	0.484	18.2
8		1627.0	16.3 (0.74)	0.262	9.6
*9*		*1617.7*	*21.2 (0.77)*	*0.210*	
*10*		*1613.1*	*29.7 (0.49)*	*0.019*	
*11*		*1579.0*	*15.6 (0.00)*	*0.056*	
***Co-fitting (weight: 300)***					
1	Same	1701.1	12.7 (0.52)	0.019	0.5
2	as	1688.5	18.7 (0.98)	0.084	3.0
3	above	1672.8	27.5 (0.84)	0.323	17.8
4		1660.1	22.5 (0.50)	0.340	17.5
5		1652.7	15.3 (0.70)	0.371	12.0
6		1644.1	18.7 (0.40)	0.398	17.6
7		1636.4	18.4 (0.73)	0.472	18.2
8		1626.9	18.6 (0.70)	0.339	13.4
*9*		*1616.0*	*18.4 (0.68)*	*0.107*	
*10*		*1612.1*	*26.1 (0.69)*	*0.056*	
*11*		*1581.5*	*18.8 (0.91)*	*0.075*	

Concomitant fitting of the absorption and second derivative spectra using a weight of 30 yields the results shown in Figure 4B. In contrast to the previous case, the second derivative of the fit shows a strong superimposition throughout the fitting range and, in particular, within the region of complex overlap that had not been modelled properly in the previous fit. The plot of the residuals between the fitted and experimental absorption spectra has a smoother appearance in the central region of the fitted spectral range but reveals slight distortions towards the edges of the fitting range. Altogether, this results in an SD_Abs_ of 2.2 × 10^−3^, a value higher than in the case of fitting the absorption spectrum only. However, the fit in the central region of the amide I band is improved as observed above for co-fitting of the ribonuclease A spectrum: the SD_Abs_ calculated within the 1700–1600 cm^−1^ interval decreases from a value of 1.15 × 10^−3^ for standard fitting to a value of 4.17 × 10^−4^ for co-fitting. This indicates again that co-fitting decreases SD_Der_ at the expense of SD_Abs_ when the entire fitting range is considered, but it improves the fit to both absorption and second derivative spectrum in those regions where second derivative bands are most intense. The particular benefit in this case was a better modelling of a region containing extensively overlapping bands.

To test the effect of increasing weights for the co-fitting, this procedure was repeated using a weight of 300, a value one order of magnitude higher than the previous one. The fit results are shown in Figure 4C. The second derivative spectrum of the fit is, as in the case of a weight of 30, nearly indistinguishable from the experimental spectrum, which would suggest that increasing the weight has a marginal effect on the quality of the fit (the SD_Der_ decreases by 38% to 7.45 × 10^−5^). However, the agreement between absorption spectrum and fit is much worse. The SD_Abs_ is, with a value of 9.9 × 10^−3^, significantly higher than in the two previous cases. The plot of the residuals has a largely distorted appearance throughout the fitting range. This decrease in quality of the fit to the absorption spectrum was observed when the weighted second derivative had approximately the same amplitude as the absorption spectrum. Thus, forcing the agreement between the second derivatives of the fitted and experimental spectra can have detrimental consequences on the agreement between the corresponding absorption spectra. Further confirmation for this interpretation was obtained using a weight of 3000 for co-fitting (data not shown) which, as expected, decreased the quality of the fit to the absorption spectrum even further (SD_Abs_ 3.42 × 10^−2^) in favor of the fit to the second derivatives (SD_Der_ 4.73 × 10^−5^).

Visual inspection of the agreement between the second derivatives of the fitted and the experimental spectrum may, therefore, not be sufficient to select the best fit. The analysis of the second derivatives shown in Figure 4, panels B and C, would, for instance, suggest that the fits yield similar results, with the SD_Der_ displaying relatively similar values (1.2 × 10^−4^ and 7.45 × 10^−5^). The overall characteristics of the component bands detected are, however, different. With a weight of 300, for instance, the intense α-helix band at 1657 cm^−1^ decreases in favor of turns (1671 cm^−1^). Interestingly, the band areas obtained with a weight of 300 are very similar to the standard fitting approach although most of the bands are considerably narrower. The largest difference between the weight 30 fit and the other fits in terms of estimated secondary structure content is the higher α-helix and irregular structure content found by the former (46% *vs.* 25%–30%) which is in better agreement with the secondary structure content from X-ray crystallography (38% helix and 20% unassigned).

We note that an improvement of the agreement between the second derivatives of fit and experimental spectrum improves also the agreement of the fourth derivatives (not shown). The standard fitting approach does not reproduce any of the bands below 1650 cm^−1^ whereas this is the case for the fit with a weight of 30. Increasing the weight to 300 gives a nearly perfect fit even of the small band at 1663 cm^−1^.

#### 2.4.2. Aconitase

The second and fourth derivative spectra of aconitase show an unexpectedly simple line shape for the amide I band, consisting mainly of a strong band at 1656 cm^−1^ (α-helices), a band at 1641 cm^−1^ and a weaker band at 1625 cm^−1^ (β-sheets), and two weak bands at 1692 cm^−1^ (high-frequency component of anti-parallel β-sheets) and 1677 cm^−1^ (turns). The weak, sharp band at 1614 cm^−1^ most likely arises from amino acid side chain vibrations, as described above.

**Figure 5:**
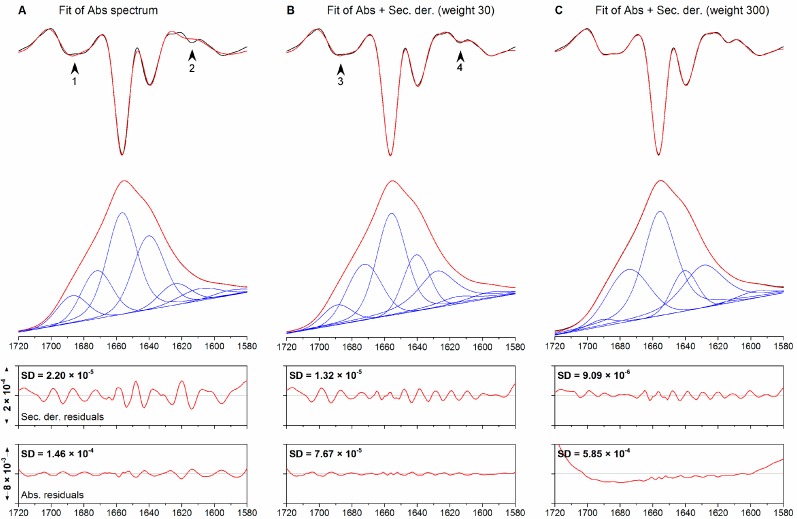
Fitting of the absorption spectrum of aconitase. (**A**) fitting of the absorption spectrum only; (**B**,**C**) simultaneous fitting of the absorption and second derivative spectra with weights of 30 and 300, respectively. From top to bottom: second derivative, absorption spectra and plots of the residuals. Original spectra are shown black, fitted spectra in red. The individual bands are shown in blue. Arrow heads denote noteworthy discrepancies between the experimental and fitted spectra.

Standard fitting of the aconitase spectrum leads to the low SD_Abs_ value of 1.46 × 10^−4^ (Figure 5A). Band parameters are reported in Table 4. The second derivatives of the fit and of the experimental spectrum appear to be, by visual inspection, relatively well superimposed, with the exception of the band at 1614 cm^−1^ (arrowhead 2 in Figure 5A), which cannot be observed in the second derivative of the fit, and the two bands of similar intensities at 1692 and 1677 cm^−1^, which merge to give rise to a broad composite band centered at approx. 1687 cm^−1^ (arrowhead 1). However, the plot of the residuals appears to be flat within the entire fitting range.

Co-fitting using a weight of 30 reduces the SD_Abs_ by a factor of almost two (7.67 × 10^−5^, Figure 5B). A possible explanation is that in the case of co-fitting, trapping of the fit routine in local minima can be avoided by the need for agreement between fitted and experimental second derivative spectra. The second derivative of the fit nicely shows features that were absent in the previous fit, such as the individual band at 1614 cm^−1^ (arrowhead 4 in Figure 5B), and enhanced superposition of the bands at 1692 and 1677 cm^−1^ (arrowhead 3). Quantitatively, this results in a decrease of the SD_Der_ from 2.2 × 10^−5^ to 1.32 × 10^−5^.

Increasing the weight of the co-fitting to 300 further decreases the SD_Der_ (from 1.32 × 10^−5^ to 9.09 × 10^−6^). Visual comparison shows that the agreement of the second derivatives of the fit and of the experimental spectrum is good. This is true also for the absorption spectra. However, inspection of the plot of the residuals calculated on the absorption spectrum reveals significant disagreement in the turn and high-frequency β-sheet region (1700–1670 cm^−1^, and up to 1720 cm^−1^), as well as below 1600 cm^−1^.

**Table 4:** Band parameters obtained upon standard fitting and co-fitting of aconitase. Values in italics correspond to amino acid side chain absorptions and where excluded from calculation of band area of the amide I band. The fg values denotes the fractional contribution of the Gaussian lineshape to the overall lineshape. The initial value was 0.50 for all bands.

Band	Initial Position	Final Position	Width (fg)	Intensity	Area/%
***Fitting of Abs spectrum alone***					
1	1692	1687.1	22.0 (1.00)	0.049	9.3
2	1677	1672.1	22.0 (1.00)	0.086	16.5
3	1656	1656.6	21.7 (0.70)	0.182	38.9
4	1641	1640.3	22.8 (0.86)	0.132	27.9
5	1625	1624.2	22.3 (1.00)	0.038	7.4
*6*	*1614*	*1615.4*	*28.9 (0.89)*	*0.011*	
*7*	*1609*	*1609.1*	*29.5 (1.00)*	*0.021*	
*8*	*1590*	*1590.0*	*26.0 (1.00)*	*0.009*	
***Co-fitting (weight: 30)***					
1	Same	1689.1	20.8 (0.96)	0.035	6.3
2	as	1672.6	24.7 (1.00)	0.100	20.7
3	above	1655.8	21.3 (0.75)	0.182	36.4
4		1640.5	19.0 (1.00)	0.100	16.0
5		1627.8	27.8 (0.22)	0.065	20.5
*6*		*1615.7*	*28.9 (0.00)*	*0.013*	
*7*		*1609.6*	*28.5 (0.48)*	*0.021*	
*8*		*1596.2*	*27.5 (0.38)*	*0.010*	
***Co-fitting (weight: 300)***					
1	Same	1691.5	18.3 (0.61)	0.012	2.1
2	as	1675.0	30.0 (1.00)	0.093	22.0
3	above	1655.8	23.9 (0.52)	0.187	42.6
4		1640.6	18.2 (1.00)	0.073	10.4
5		1628.7	30.5 (0.42)	0.076	22.9
*6*		*1613.8*	*15.0 (0.47)*	*0.035*	
*7*		*1609.0*	*27.6 (0.27)*	*0.003*	
*8*		*1597.7*	*25.4 (1.00)*	*0.012*	

Fitting with different weights affects some of the band areas obtained: the 1690 cm^−1^ band area decreases with increasing weight (from 9% for standard fitting to 2% for co-fitting with a weight of 300). The same is true for the 1641 cm^−1^ band area which decreases (from 28% to 10%) at the expense of an increase of the ~1626 cm^−1^ band area (from 7% to 23%). Since the latter two bands are assigned to β-sheets, this difference does not significantly change a secondary structure analysis.

The main bands in the fourth derivative spectrum are already satisfactorily reproduced in the standard fitting approach. Increasing the weight to 300 improves the agreement, in particular for the bands at 1692 and 1614 cm^−1^ (not shown).

### 2.5. Fitting in the Presence of Water Vapor Bands

Water vapor exhibits several sharp and intense absorption bands encompassing the entire amide I region of proteins. This is the reason why all research-grade spectrometers, and most industry-grade spectrometers, are continuously purged with dry air or N_2_. However, spectrometer manufacturers have introduced small, unpurged instruments that are being increasingly used to analyze the secondary structure of proteins. The following section demonstrates the limitations of such an approach.

**Figure 6:**
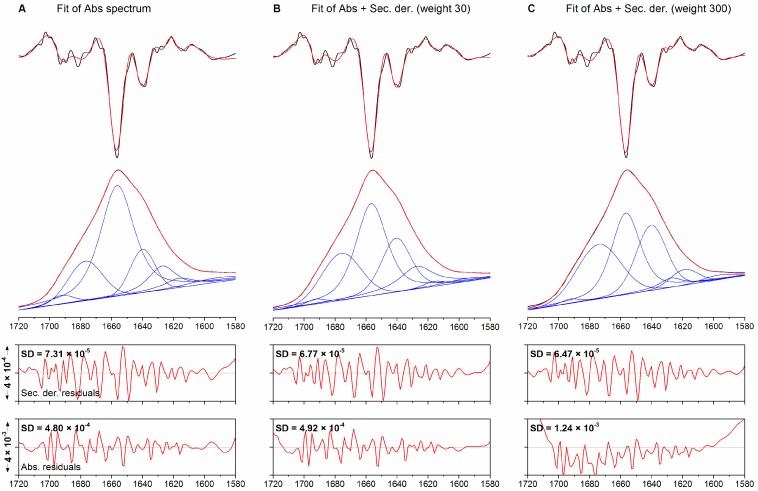
Fitting of the absorption spectrum of aconitase recorded without purging the spectrometer with dry air. (**A**) fitting of the absorption spectrum only; (**B**,**C**) simultaneous fitting of the absorption and second derivative spectra with weights of 30 and 300, respectively. From top to bottom: second derivative, absorption spectra and plots of the residuals. Original spectra are shown black, fitted spectra in red. The individual bands are shown in blue.

In theory, if sample absorption is strong relative to water vapor absorption in ambient air, and if the sample and background spectra contain water vapor signals of similar intensities, the resulting absorption spectrum will be largely devoid of water vapor bands and the amide I line shape largely unaffected. Second derivative spectra are more sensitive to water vapor contamination since band intensities in second (or fourth) derivative spectra are strongly dependent on bandwidths. Therefore, the narrow water vapor bands are enhanced considerably compared to the broader protein bands. In addition to this, it is virtually impossible for sample and background spectra to contain identical water vapor signals. Local fluctuations in temperature, pressure and air currents within the instrument continuously alter the number of water vapor molecules probed by the infrared beam. This phenomenon becomes worse in spectrometers with long beam paths, like the one used in this study, and in unpurged spectrometers used under high humidity conditions (such as on a warm summer day). Because second derivative spectra are more sensitive to water vapor contamination than absorption spectra, co-fitting of these protein spectra may lead to inaccurate results, or show lower accuracy than standard fitting. To test this hypothesis, the spectrometer was let to equilibrate with ambient humidity for several days with the dry air purge switched off. Then a background spectrum was recorded, a further aconitase film prepared on the ATR crystal, and a sample spectrum recorded. The resulting absorption spectrum (Figure 6A) does not show water vapor peaks. The second derivative, however, reveals several sharp features originating from water vapor bands. Standard fitting yields a SD_Abs_ of 4.80 × 10^−4^, a value nearly 3.5-fold higher as the one calculated for the spectrum in the absence of water vapor bands (Figure 5A).

Co-fitting using a weight of 30 leads to a very moderate improvement of the SD_Der_, and a moderate worsening of the SD_Abs_ (Figure 6B). This is in clear contrast with what is observed in the absence of water vapor, namely a significant improvement of the SD for both the absorption spectra and their second derivative.

Increasing the weight to 300 only moderately improves the SD of the second derivative with respect to a weight of 30. This is, again, in contrast with fitting and co-fitting in the absence of water vapor, where a constant improvement of the second derivative SD can be observed.

These results suggest that the presence of water vapor makes co-fitting of absorption and second derivative spectra less accurate than standard fitting. However, because co-fitting *per se* allows for better fitting and secondary structure quantification, it is essential to obtain water vapor-free spectra to achieve accurate and realistic fits.

One might argue that the water vapor effect obtained with our large spectrometer is considerably more severe than that for a small spectrometer. Indeed, the path length of the infrared beam in our spectrometer is ~130 cm, whereas we estimate it to be 40–50 cm in a small instrument. Thus, the water vapor content is about three times higher in our instrument. On the other hand, the outdoor relative humidity at the time of the measurement was 92% and the temperature 3.9 °C (data from the weather station of the Department of Meteorology of Stockholm University on the roof of our building). This corresponds to a relative humidity of ~30% in our laboratory. Therefore, the water vapor content in our large spectrometer at the time of the measurement corresponds to that of a small spectrometer on a warm and humid day. It was less than in a small spectrometer on a hot and humid day. Therefore our results are relevant for the performance of small spectrometers.

## 3. Experimental Section

### 3.1. Materials

The following protein samples were purchased from Sigma (St. Louis, MO, USA): aconitase (porcine heart), pyruvate kinase (rabbit muscle), ribonuclease A (bovine pancreas). MOPS and NaCl were from Sigma.

### 3.2. Preparation of Protein Samples

The lyophilyzed protein samples were dissolved in 50 mM MOPS, 100 mM NaCl (pH 7) at a final concentration of 2 mg·mL^−1^. Samples were incubated at room temperature until complete dissolution, and immediately subjected to the infrared measurements.

### 3.3. Infrared Spectroscopy

Infrared absorption spectra were recorded by means of a Vertex 70 Fourier-transform infrared spectrometer (Bruker Optics, Ettlingen, Germany) continuously purged with CO_2_-free, dry air and equipped with a 9-reflection DuraSampl*IR* II diamond ATR accessory (SensIR, North Brunswick, NJ, USA). Interferograms were recorded at a resolution of 4 cm^−1^, apodized using a Blackman-Harris 3-term apodization function and Fourier-transformed with a zero-filling factor of 2. Five hundred scans were averaged and stored as a background spectrum. To record spectra of hydrated protein films, 5 µL (10 µg protein) from each protein sample were uniformly spread onto the crystal and dried under a gentle stream of N_2_ at room temperature. Twenty spectra, each averaged over 150 scans, were recorded during evaporation of the bulk aqueous solution. The last ten spectra from each series, originating from a stable hydrated film, were averaged. Three independent experiments were performed for each protein sample.

### 3.4. Spectral Analysis and Curve-Fitting

Preliminary spectral analyses were performed with the OPUS software from the instrument manufacturer. They include calculation of the second and fourth derivative spectra in Figure 2, which were calculated in a smoothing window of nine datapoints by the Savitzky-Golay algorithm. Subsequent analyses and curve-fitting were performed with the Kinetics software written by E. G. running in a MATLAB environment. Fitting of the amide I absorption spectra was performed between 1720 and 1580 cm^−1^. Component bands were detected upon analysis of fourth derivative spectra. During fitting, the final position of each band was allowed to explore a 10 cm^−1^ window centered around its initial position. The line shape was a weighted average of a Gaussian and a Lorentzian line. The fractional contribution of the Gaussian line shape was 0.5 at the beginning of the fit. A straight, dynamic (e.g., recomputed at each round of fitting) line was drawn between the fitting extremes. It describes a quasi -linear contribution that can include the tail of several bands located outside the fitting range as well as a superimposition of a series of weak contributions throughout the fitting range.

For co-fitting of both absorption and second derivative spectra, the latter was multiplied by factors of 30, 300, and 3000 to compensate for the lower intensity of the latter with respect to the former. The initial band parameters (position, width and intensity), including the number of bands, were the same as for fitting to the absorption spectra only. The co-fitting option was implemented in a pre-existing version of Kinetics. Also with this program, the second derivative was calculated using nine data points and the Savitzky-Golay algorithm. To avoid truncation effects, the second derivative was calculated over a larger spectral range than the range used for assessing the quality of the fit. At both ends, the range is extended by the number of data points used for calculation of the second derivative. In our case the second derivative was calculated between 1729 and 1571 cm^−1^, whereas the fit quality was assessed between 1720 and 1580 cm^−1^.

Curve fitting was achieved by the “fmincon” Matlab function without gradient or Hessian information as input. The function performs a constrained nonlinear optimization that finds a constrained minimum of a scalar function of several variables starting at an initial estimate. The maximum number of iterations (MaxFunEvals) was set to 100× the number of variables. Optimization was also stopped when the predicted change in the objective function (TolFun) was less than 10^−6^. At each iteration, the bands and their second derivatives were calculated and used together to fit simultaneously the spectrum and its second derivative.

### 3.5. Secondary Structure Analysis

The secondary structure content in each protein structure was obtained by analysis of the corresponding PDB file with the DSSP algorithm (The Secondary Structure Server, http://2struc.cryst.bbk.ac.uk/twostruc). Secondary structures in infrared spectra were assigned according to values reported elsewhere in the literature [2,3,40,41]. Briefly, signals between 1641 and 1620 cm^−1^ were assigned to β-sheets. The presence of a sharp, but weaker (approx. one fifth in intensity) band between 1695 and 1680 cm^−1^ allows to discriminate between the parallel and the antiparallel arrangement of the strands within the β-sheets. Signals between 1680 and 1660 cm^−1^ were assigned to β-turns or turn-like structures and signals between 1660 and 1650 cm^−1^ to α-helices and irregular conformations. The latter are additionally thought to cause bands between 1650 and 1642 cm^−1^. Signals below 1615 cm^−1^ usually arise from amino acid side chains. Quantitative analysis of secondary structure content in infrared spectra was performed by curve-fitting with Kinetics as reported in the previous paragraph.

## 4. Conclusions

Determination of band parameters in the crowded infrared spectra of proteins often represents a bottleneck towards the use of infrared spectroscopy in the study of protein structures and conformations. Curve fitting, a procedure involving reconstruction of an experimental spectrum given a defined set of initial component bands, is the most widely used approach for qualitative and quantitative studies of proteins with infrared spectroscopy. However, a good fit of the absorption spectrum does not necessarily imply that the fitted component bands are a good model for the experimental spectrum [3]. The quality of the fit model should be checked by comparing the resolution-enhanced fitted spectrum. If these two spectra disagree, the fit is clearly not a correct description of the experimental data. Therefore, we propose an extension of the standard curve fitting approach based on concomitant fitting of an absorption spectrum and its second derivative (co-fitting).

Co-fitting makes best use of the information that is encoded in the spectrum. It allows identification and correct modelling of weaker bands and other minor features within or near the amide I region (Figure 3, arrowheads), providing a more accurate modelling of the experimental data. Co-fitting is particularly valuable in crowded spectral regions, as the region of β-sheet absorption of pyruvate kinase (Figure 4). However, it also improves the fit result for the simpler spectra of ribonuclease A and aconitase, in particular in the center of the amide I region, where the amplitude of the second derivative spectra is large. From visual inspection, it might be argued that this improvement is marginal, but the effects on band areas of the fitted component bands can be quite dramatic. This shows that every effort should be made to model all properties of the spectrum, including its second derivative. Co-fitting introduces additional constraints for the fit and therefore leads to a better description all the features that are contained in the experimental spectrum.

To some extent, performing curve fitting can be compared to folding a protein in a potential energy landscape. The native conformation (absolute minimum) of a globular protein often has an energy value separated from aberrant conformations (local minima) by the equivalent of a few hydrogen bonds. In a similar way, curve fitting may end in a local minimum in the “difference-from-experiment” landscape; the fit appears to be similar to the experimental spectrum, but fails to model the actual band pattern. Inclusion of additional constraints, like an agreement between the second derivatives of fit and experimental spectrum may avoid trapping of the fit routine in a local minimum. Such a behavior was observed in the case of aconitase.

Our results show that visual agreement between the second derivatives of the fit and of the original spectrum is a quick tool to assess the quality of the fit, but that it is beneficial to explore different weight factors so that the lowest possible standard deviation is obtained. In our hands, multiplying the second derivative spectrum with a factor of 30 gave the best results. This factor compensates only partially for the 300-fold smaller amplitude of second derivative spectra compared to the absorption spectra. On the other hand, the discrepancy between fit and experiment is larger for the second derivatives than for the absorption spectra. If the fits to the second derivatives were equally good as those to the absorption spectra, one would expect that the residuals are 300-times smaller. But they are only 10-times smaller at the optimum weight. This means that normalized to the maximum amplitude in the spectra, the residuals of the second derivatives are 30-times larger than those of the absorption spectra. Thus the difficulty of fitting the second derivatives implies an enhanced influence of the second derivatives on the outcome of the fit even at low weights.

Increasing the weight of the second derivative in the fit improves also the agreement between the fourth derivatives of fit and experimental spectrum. Therefore it seems that the fourth derivative does not contain additional information and that it is sufficient to include only the second derivative and not the fourth derivative in the co-fitting approach. The second derivative has the advantage of being less affected by noise and water vapor than the fourth derivative.

Thus, including the second derivative improves the description of the complex contour of the amide I absorption. On the other hand, the agreements of the fit to the absorption spectrum and to the second derivative spectrum need to be balanced carefully, as too strong an emphasis on the second derivative spectrum deteriorates the agreement with the absorption spectrum. This effect indicates that the fitted component bands still do not exactly represent those that build up the experimental amide I band. Another indication for this is that even in the presence of co-fitting, the agreement between the second derivatives of fit and experimental spectrum is not perfect. This could point to additional component bands that need to be included in the fit. These additional components can only be identified when co-fitting is used, not when only the absorption spectrum is fitted. In this work, we refrain from adding more bands since our aim was to provide a comparison with the standard approach using the same initial fit parameters for fits with and without co-fitting.

Co-fitting affects the relative band areas of the fit, which are the basis for an estimation of the secondary structure. For two of the studied proteins, ribonuclease A and pyruvate kinase, co-fitting improves a quantification of the secondary structure content with respect to standard fitting. For the third protein, there was little change. We do not want to put too much emphasis on this observation, since our conditions were optimized for obtaining high quality spectra, whereas the preparation of protein films might modify the secondary structure content found in solution. A detailed secondary structure analysis would also shift the focus away from the point that we want to make, which is that co-fitting provides a better modelling of the experimental data and that the band parameters yielded by this approach are, therefore, likely to be more accurate than those obtained by standard fitting.

A prerequisite of co-fitting are spectra of highest quality so that the second derivative spectrum is free from e.g., water vapor contributions. For this, the spectrometer should be purged with dry air or N_2_. While this has been known for a long time, the authors have the impression that this knowledge has been partially lost in the field and that it is not conveyed by the sales representatives of spectrometer manufacturers. Therefore we have included a clear demonstration that unpurged spectrometers are unsuitable for a detailed analysis of protein secondary structure (Figure 6). Such analysis involves resolution enhanced spectra, such as second derivative spectra, which are very sensitive to water vapor contamination. This is true even when co-fitting is not used. When it is used with spectra contaminated by water vapor, the benefits of the approach are lost and turned into a disadvantage.

In conclusion, simultaneous fitting of an absorption spectrum and of its second derivative can lead to considerable improvements in the accuracy and reliability of band parameters and provides a more robust base for secondary structure quantification in infrared spectra of proteins.

## Abbreviations

SD: standard deviation
SD_Abs_: standard deviation of the difference between two absorption spectra
SD_Der_: standard deviation of the difference between two second derivative spectra

## References

[1] Yang H., Yang S., Kong J., Dong A., Yu S. (2015). Obtaining information about protein secondary structures in aqueous solution using Fourier transform infrared spectroscopy. Nat. Protoc..

[2] Barth A. (2007). Infrared spectroscopy of proteins. Biochim. Biophys. Acta Bioenerg..

[3] Barth A., Zscherp C. (2002). What vibrations tell about proteins. Q. Rev. Biophys..

[4] Mäntele W. (2015). The analysis of protein conformation by infrared spectroscopy: An introduction of the editor to a scientific dispute. Spectrochim. Acta Part A Mol. Biomol. Spectrosc..

[5] Manning M.C. (2005). Use of infrared spectroscopy to monitor protein structure and stability. Expert Rev. Proteomics.

[6] Gilmanshin R., Williams S., Callender R.H., Woodruff W.H., Dyer R.B. (1997). Fast events in protein folding: Relaxation dynamics of secondary and tertiary structure in native apomyoglobin. Proc. Natl. Acad. Sci. USA.

[7] Williams S., Causgrove T.P., Gilmanshin R., Fang K.S., Callender R.H., Woodruff W.H., Dyer R.B. (1996). Fast Events in Protein Folding: Helix Melting and Formation in a Small Peptide. Biochemistry.

[8] Heimburg T., Schünemann J., Weber K., Geisler N. (1999). FTIR-Spectroscopy of Multistranded Coiled Coil Proteins. Biochemistry.

[9] Karjalainen E.L., Barth A. (2012). Vibrational Coupling between Helices Influences the Amide I Infrared Absorption of Proteins: Application to Bacteriorhodopsin and Rhodopsin. J. Phys. Chem. B.

[10] Karjalainen E.L., Ravi H.K., Barth A. (2011). Simulation of the Amide I Absorption of Stacked β-Sheets. J. Phys. Chem. B.

[11] Choi J.H., Ham S., Cho M. (2002). Inter-peptide interaction and delocalization of amide I vibrational excitons in myoglobin and flavodoxin. J. Chem. Phys..

[12] Strasfeld D.B., Ling Y.L., Gupta R., Raleigh D.P., Zanni M.T. (2009). Strategies for Extracting Structural Information from 2D–IR Spectroscopy of Amyloid: Application to Islet Amyloid Polypeptide. J. Phys. Chem. B.

[13] Schweitzer-Stenner R., Measey T.J. (2010). Simulation of infrared, Raman and VCD amide I band profiles of self-assembled peptides. Spectroscopy.

[14] Chehín R., Iloro I., Marcos M.J., Villar E., Shnyrov V.L., Arrondo J.L. (1999). Thermal and pH-induced conformational changes of a β-sheet protein monitored by infrared spectroscopy. Biochemistry.

[15] Herman P., Staiano M., Marabotti A., Varriale A., Scirè A., Tanfani F., Vecer J., Rossi M., D’Auria S. (2006). D-Trehalose/D-maltose-binding protein from the hyperthermophilic archaeon Thermococcus litoralis: The binding of trehalose and maltose results in different protein conformational states. Proteins Struct. Funct. Bioinform..

[16] Abbott G.W., Ramesh B., Srai S.K. (2012). Interaction between Soluble and Membrane-Embedded Potassium Channel Peptides Monitored by Fourier Transform Infrared Spectroscopy. PLoS ONE.

[17] Adato R., Altug H. (2013). *In situ* ultra-sensitive infrared absorption spectroscopy of biomolecule interactions in real time with plasmonic nanoantennas. Nat. Commun..

[18] Barth A., Zscherp C. (2000). Substrate binding and enzyme function investigated by infrared spectroscopy. FEBS Lett..

[19] Baldassarre M., Scirè A., Tanfani F. (2012). Turning pyridoxal-5'-phosphate-dependent enzymes into thermostable binding proteins: D-Serine dehydratase from baker’s yeast as a case study. Biochimie.

[20] Dodd J.G., DeNoyer L.K., Chalmers J.M., Griffiths P.R. (2002). Handbook of Vibrational Spectroscopy.

[21] Arrondo J.L., Muga A., Castresana J., Goñi F.M. (1993). Quantitative studies of the structure of proteins in solution by Fourier-transform infrared spectroscopy. Prog. Biophys. Mol. Biol..

[22] Byler D.M., Wilson R.M., Randall C.S., Sokoloski T.D. (1995). Second derivative infrared spectroscopy as a non-destructive tool to assess the purity and structural integrity of proteins. Pharm. Res..

[23] Susi H., Byler D.M. (1983). Protein structure by Fourier transform infrared spectroscopy: Second derivative spectra. Biochem. Biophys. Res. Commun..

[24] Kauppinen J.K., Moffat D.J., Mantsch H.H., Cameron D.G. (1981). Fourier self-deconvolution: A method for resolving intrinsically overlapped bands. Appl. Spectrosc..

[25] Lórenz-Fonfría V.A., Padrós E. (2004). Curve-fitting of Fourier manipulated spectra comprising apodization, smoothing, derivation and deconvolution. Spec. Acta Part A.

[26] Holler F., Burns D.H., Callis J.B. (1989). Direct Use of Second Derivatives in Curve-Fitting Procedures. Appl. Spectrosc..

[27] Gasda P.J., Ogliore R.C. (2014). Modeling the Raman spectrum of graphitic material in rock samples with fluorescence backgrounds: Accuracy of fitting and uncertainty estimation. Appl. Spectrosc..

[28] Dong A., Huang P., Caughey W.S. (1990). Protein secondary structures in water from second-derivative amide I infrared spectra. Biochemistry.

[29] Shen X., Li H., Ou Y., Tao W., Dong A., Kong J., Ji C., Yu S. (2008). The Secondary Structure of Calcineurin Regulatory Region and Conformational Change Induced by Calcium/Calmodulin Binding. J. Biol. Chem..

[30] Singh B.R., Singh B.R. (1999). Basic Aspects of the Technique and Applications of Infrared Spectroscopy of Peptides and Proteins. Infrared Analysis of Peptids and Proteins Principles and Applications.

[31] Mikhailyuk I.K., Lokstein H., Razjivin A.P. (2005). A method of spectral subband decomposition by simultaneous fitting the initial spectrum and a set of its derivatives. J. Biochem. Biophys. Methods.

[32] Li C., Kumar S., Montigny C., le Maire M., Barth A. (2014). Quality assessment of recombinant proteins by infrared spectroscopy. Characterisation of a protein aggregation related band of the Ca^2+^-ATPase. Analyst.

[33] Wlodawer A., Bott R., Sjölin L. (1982). The refined crystal structure of ribonuclease A at 2.0 Å resolution. J. Biol. Chem..

[34] Larsen T.M., Laughlin L.T., Holden H.M., Rayment I., Reed G.H. (1994). Structure of Rabbit Muscle Pyruvate Kinase Complexed with Mn^2+^, K^+^, and Pyruvate. Biochemistry.

[35] Lloyd S.J., Lauble H., Prasad G.S., Stout C.D. (1999). The mechanism of aconitase: 1.8 Å resolution crystal structure of the S642A: Citrate complex. Protein Sci..

[36] Krimm S., Bandekar J. (1986). Vibrational spectroscopy and conformation of peptides, polypeptides, and proteins. Adv. Protein Chem..

[37] Olinger J.M., Hill D.M., Jakobsen R.J., Brody R.S. (1986). Fourier transform infrared studies of ribonuclease in H2O and 2H2O solutions. Biochim. Biophys. Acta.

[38] Barth A. (2000). The infrared absorption of amino acid side chains. Prog. Biophys. Mol. Biol..

[39] Yu S., Lee L.L.-Y., Ching Lee J. (2003). Effects of metabolites on the structural dynamics of rabbit muscle pyruvate kinase. Biophys. Chem..

[40] Surewicz W.K., Mantsch H.H., Chapman D. (1993). Determination of protein secondary structure by transform infrared spectroscopy: A critical assessment. Biochemistry.

[41] Jackson M., Mantsch H.H. (1995). The Use and Misuse of FTIR Spectroscopy in the Determination of Protein Structure. Crit. Rev. Biochem. Mol. Biol..

